# Kernel Density Estimation of Electromyographic Signals and Ensemble Learning for Highly Accurate Classification of a Large Set of Hand/Wrist Motions

**DOI:** 10.3389/fnins.2022.796711

**Published:** 2022-03-09

**Authors:** Parviz Ghaderi, Marjan Nosouhi, Mislav Jordanic, Hamid Reza Marateb, Miguel Angel Mañanas, Dario Farina

**Affiliations:** ^1^The Biomedical Engineering Department, Engineering Faculty, University of Isfahan, Isfahan, Iran; ^2^Laboratory of Sensory Processing, Brain Mind Institute, Faculty of Life Sciences, École Polytechnique Fédérale de Lausanne (EPFL), Lausanne, Switzerland; ^3^Biomedical Engineering Research Centre (CREB), Automatic Control Department (ESAII), Universitat Politècnica de Catalunya-Barcelona Tech (UPC), Barcelona, Spain; ^4^CIBER de Bioingeniería, Biomateriales y Nanomedicina (CIBER-BBN), Madrid, Spain; ^5^Department of Bioengineering, Imperial College London, London, United Kingdom

**Keywords:** electromyography, ensemble learning, kernel density estimation, machine learning, myoelectric control, prosthetics

## Abstract

The performance of myoelectric control highly depends on the features extracted from surface electromyographic (sEMG) signals. We propose three new sEMG features based on the kernel density estimation. The trimmed mean of density (TMD), the entropy of density, and the trimmed mean absolute value of derivative density were computed for each sEMG channel. These features were tested for the classification of single tasks as well as of two tasks concurrently performed. For single tasks, correlation-based feature selection was used, and the features were then classified using linear discriminant analysis (LDA), non-linear support vector machines, and multi-layer perceptron. The eXtreme gradient boosting (XGBoost) classifier was used for the classification of two movements simultaneously performed. The second and third versions of the Ninapro dataset (conventional control) and Ameri’s movement dataset (simultaneous control) were used to test the proposed features. For the Ninapro dataset, the overall accuracy of LDA using the TMD feature was 98.99 ± 1.36% and 92.25 ± 9.48% for able-bodied and amputee subjects, respectively. Using ensemble learning of the three classifiers, the average macro and micro-F-score, macro recall, and precision on the validation sets were 98.23 ± 2.02, 98.32 ± 1.93, 98.32 ± 1.93, and 98.88 ± 1.31%, respectively, for the intact subjects. The movement misclassification percentage was 1.75 ± 1.73 and 3.44 ± 2.23 for the intact subjects and amputees. The proposed features were significantly correlated with the movement classes [Generalized Linear Model (GLM); *P*-value < 0.05]. An accurate online implementation of the proposed algorithm was also presented. For the simultaneous control, the overall accuracy was 99.71 ± 0.08 and 97.85 ± 0.10 for the XGBoost and LDA classifiers, respectively. The proposed features are thus promising for conventional and simultaneous myoelectric control.

## Introduction

The electromyographic (EMG) signal is the electrical manifestation of the neuromuscular activation when muscles contract (Raez et al., 2006). The non-invasive EMG (surface EMG, sEMG) finds applications in rehabilitation (Parker et al., 2006), sport science (Clarys, 2000), kinesiology and ergonomics (Hogrel, 2005), muscle architecture identification (Marateb et al., 2016), neuromuscular pathology (Haig et al., 1996), and neurological disease diagnosis (Zwarts et al., 2000). Among the applications in rehabilitation technology, the myocontrol of active upper limb prostheses has a relevant clinical impact. For this purpose, sEMG provides information on the neural control of the motor intention of the users (Jordanic et al., 2016). However, although current robotic technology has reached a satisfactory level, EMG-based controlling schemes are still coarse.

One paradigm for myoelectric control is pattern recognition. Pattern recognition systems are trained to recognize patterns from the sEMG and select the corresponding function to execute. This control scheme assumes that sEMG features recorded from a given electrode location are repeatable for a given state of muscle group activation and are different from one state of activation to another (Ameri et al., 2014).

In sEMG pattern recognition research, several features and classifiers have been tested. For example, Zardoshti-Kermani et al. (1995) evaluated a feature extraction method called histogram and proved that its accuracy for classifying three movements was approximately 95%. Boostani and Moradi (2003) selected the best feature set from 19 features that included wavelet coefficients to classify 15 movements and observed that the energy of wavelet coefficients and the cepstrum coefficients led to the best performance. Yonghong and Englehart used a Gaussian Mixture Model (GMM) and the combination of Root-Mean-Square (RMS) and Auto-Regressive (AR) features to discriminate between six hand movements with an average accuracy of 96.26%. Atzori et al. systematically evaluated seven feature sets and five classifiers on 27 intact subjects performing 52 hand, wrist, and forearm movements. They showed that simple time-domain features, as proposed originally by Hudgins et al. (1993), performed as well as complex and computationally more demanding features, such as marginal discrete wavelet transform (mDWT) and Short-Time Fourier Transform (STFT). Nonetheless, for 52 movements, the classification accuracy was below 80% in the best case (Atzori et al., 2015). AbdelMaseeh et al. (2016) extracted multi-channel EMG activation trajectories and classified the extracted trajectories using a metric based on multi-dimensional dynamic time warping. They evaluated their proposed method on the second version of the NinaPro dataset, with an overall accuracy of 89% for classifying 40 movements. Ameri et al. applied a customized transfer learning method based on Convolutional Neural Network (CNN), pretrained without electrode shift, and tested on 2.5 cm electrode displacement during flexion-extension and pronation-supination. Using ten channels, the error rate of 21.5 ± 2.3% and 46.0 ± 4.1% were obtained for flexion-extension and pronation-supination (Ameri et al., 2020). Marano et al. (2021) showed that inter- and intra-subject domain adaptation does not significantly improve movement classification accuracy in NinaPro DB2, DB3, and DB6 datasets regardless of the transfer learning method. Triwiyanto et al. (2020) optimized CNN’s hyperparameters, such as the number of convolution layer filters, type of optimizer, and dropout and achieved an accuracy ranging 77 and 93% for 10-class gesture classification in different motion ranges. Sri-Iesaranusorn et al. (2021) used a feed-forward deep neural network model for the classification of 41 hand and wrist movements of DB5, and DB7 (Ninapro project), and achieved an overall accuracy of 93.87 ± 1.49 and 91.69 ± 4.68%, respectively.

In addition to the above representative examples, there have been many more attempts to compare the efficacy of various feature sets (Hudgins et al., 1993; Englehart et al., 1999) or classifiers (Atzori et al., 2015, 2016). Scheme and Englehart (2011) provided a comprehensive review of pattern recognition methods for myoelectric control. In general, few novel features have been proposed with respect to the classic time-domain features, and when compared to the time-domain features, new features usually did not substantially improve the performance. With these approaches, classification accuracy is usually reported as approximately 95% for up to 10 classes. However, it decreases substantially with increasing the number of classes.

Results on EMG pattern recognition are mostly reported for able-bodied individuals, with relatively few evaluations on amputees. Because of stump conditions, the performance of EMG pattern recognition usually reduces substantially for amputees with respect to able-bodied subjects. For example, Atzori et al. (2014a) evaluated their benchmark on 11 hand-amputated subjects and reported an average classification accuracy for 50 movements of <50%, which did not improve when using deep learning (Atzori et al., 2016).

This study proposes new sEMG features to enhance the classification accuracy for a high number of hand movements. We present three new features based on the Kernel Density Estimation (KDE) of sEMG signals used for ensemble learning. Using these features on 40 intact subjects and 11 amputees for discriminating 40 movements, we prove a substantial improvement with respect to the state-of-the-art. Moreover, because daily activities require simultaneous control of more than one movement (Iqbal et al., 2018), we further tested the performance of the proposed features in detecting simultaneous movements.

The paper is organized as follows: in the next section, the experimental protocol, the pattern recognition methods used in this study, and the validation framework are presented. Section Results describes the results of the proposed movement detection algorithm. Finally, the conclusion is summarized in Section Discussion.

## Materials and Methods

### Ninapro Database

#### Ninapro V.2

**FIGURE 1:**
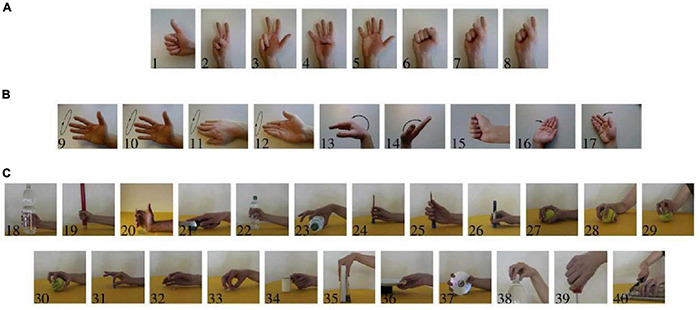
Forty movements included in the acquisition protocol. **(A)** Isometric, isotonic hand configurations (“hand postures”). **(B)** Basic movements of the wrist. **(C)** Grasping and functional movements [modified from Atzori et al. (2014a), with permission].

The second version of the Ninapro database was used for reporting results on conventional myoelectric control. This dataset provided a benchmark for hand prosthesis evaluation on intact subjects (Atzori et al., 2014a,^2015^; Gijsberts et al., 2014). The database consists of recordings with 12 sEMG wireless double-differential (DD) electrodes (DelsysTringo Wireless System; OttoBock MyoBock 13E200 sEMG electrodes) at the sampling rate of 2 ksps, with the baseline noise <750 nV RMS. Eight DD electrodes were equally spaced around the forearm at the level of the radio-humeral joint; two electrodes were placed on the main activity spots of the flexor digitorum superficialis and the extensor digitorum superficialis, and the last two electrodes were located on the main activity spots of the biceps brachii and of the triceps brachii. The main activity spots were identified by palpation (Gijsberts et al., 2014). This database contained data obtained from 40 intact subjects (70% men, 85% right-handed; age 29.9 ± 3.9 years; weight 70.9 ± 14.2 kg; height 172.8 ± 10.4 cm). The subjects were seated at a desk during the acquisitions, resting their arm comfortably on a desk. A laptop provided visual stimuli in front of the subject while recording the sEMG signals. Each subject performed six repetitions of 40 hand movements (Figure 1). Each movement lasted 5 s followed by 3 s rest.

#### Ninapro V.3

The third Ninapro database (Atzori et al., 2014a) contains data obtained from 11 trans-radial amputated subjects (11 males; 10 right-handed, one left-handed; age 42.36 ± 11.96 years; weight 79 ± 8.4 kg; height 176.9 ± 5.7). This dataset provided a benchmark for hand prosthesis evaluation on amputees (Atzori et al., 2014a,b). Amputees were asked to think to repeat the movements as naturally as possible with the missing limb. It is important to remark that amputees cannot, in general, produce reliable ground truth. In related literature, this fundamental problem has been circumvented either by (a) instructing the subjects to execute a task bilaterally while recording the ground truth from the intact limb or by (b) asking them to follow a visual stimulus (to think to repeat) (either on a screen or performed by the experimenter) (Nielsen et al., 2011). There is no consensus on the best procedure, so each subject was left free to choose after a short training phase, which resulted in only two subjects undergoing bilateral execution. As a result, the database contains only the stimulus as ground truth for the rest of the amputees. Analyses with the stimulus as ground truth have already been successfully performed [for example, see ref. Nielsen et al. (2011)]. The experimental protocol was the same as the intact subjects for the other details (Atzori et al., 2014a). Since the data for the first amputee was only available in 29 movements, it was excluded from the analysis resulting in 10 total amputees.

All intact subjects and amputees gave informed consent to the experimental procedure. The experimental protocol was approved by the Ethics Commission of the Canton Valais (Switzerland) and conformed to the Declaration of Helsinki (Atzori et al., 2014a).

### Simultaneous Movement Detection

In addition to the Ninapro database, a database recorded by Ameri et al. (2018) was used to report the performance of proposed features on the detection of simultaneous movements. This database was obtained from 17 intact subjects (15 right-handed; age 29.5 ± 3.7 years). The sEMG signals were recorded with eight bipolar electrodes, which were equally spaced around the forearm. The sampling rate of data was 1.2 kHz. Four movements, including flexion, extension, pronation, supination, and simultaneous combinations of two movements, were considered in this data set. Four repetitions of 3 s were collected for each motion (Ameri et al., 2018).

### Pre-processing

For Ninapro datasets, signals were digitally band-pass filtered using a fourth-order Butterworth filter with 20 and 500 Hz cut-off frequencies. The signal in each channel was then standardized to have zero mean with unit standard deviation. The outliers were detected in each channel using the 3-sigma rule and then removed from the dataset. Windowing was performed on the signal with a fixed size of 400 ms and an overlap of 100 ms (Gijsberts et al., 2014).

Signals collected by Ameri were filtered using an eight-order Butterworth band-pass filter with cut-off frequencies of 5 and 500 Hz. The feature extraction was based on windows of 160 ms with an increment of 40 ms (Ameri et al., 2018).

### Feature Extraction

The proposed features were calculated based on the Kernel Density Estimation (KDE) of each channel’s time samples of sEMG signals. The application of KDE in movement detection was inspired by the application of histogram, with a predefined number of bins, as proposed by Zardoshti-Kermani et al. (1995). The KDE extends the Histogram method, as described in the following.

#### Kernel Density Estimation

Density estimation is an essential tool in statistics and machine learning. It is divided into parametric and non-parametric methods. Parametric density estimation refers to methods in which the data are drawn from one of the known distribution families, and the goal of estimation is finding the parameters of the distribution from the given data (Silverman, 1986). On the other hand, there is no assumption about data distribution in the non-parametric approach (Botev et al., 2010). The histogram is the simplest non-parametric density estimation method. It is highly dependent on the number of bins and bin edges.

Moreover, it is not possible to estimate its derivatives. The KDE was proposed to overcome these drawbacks (Silverman, 1986). The task of KDE is to compute a density estimate based on *n* i.i.d. samples x_1_…x_*n*_ ∈ R drawn from an unknown density function *f*:


(1) $$\hat{\text{f}}\left(x\right)=\frac{1}{\text{nh}}\sum_{i=1}^{n}\text{K}\left(\frac{\text{x}-{\text{x}}_{i}}{\text{h}}\right)$$

where *K* is the kernel function, such that K(u) ≥ 0 and h ∈ R is the bandwidth parameter of the kernel. The selection of an appropriate kernel function and its bandwidth parameter has been discussed in the literature (Heidenreich et al., 2013).

The most common data-driven bandwidth selection technique, the plug-in method (Jones et al., 1996), is dependent on the normal reference rule. Moreover, the popular Gaussian kernel density estimator lacks local adaptivity and is sensitive to outliers. Also, most kernel estimators suffer from boundary bias when, for example, the data is non-negative (Botev et al., 2010). Botev et al. (2010) introduced an adaptive kernel density estimation method based on the smoothing properties of the linear diffusion procedure. The idea is to view the kernel from which the estimator is constructed as the transition density of a diffusion process. It incorporates information from a pilot density and then estimates the final density by the general linear diffusion procedure (Botev et al., 2010). This method has an improved plug-in bandwidth selection approach that avoids the standard reference rules deteriorating the performance of plug-in methods. Moreover, since the KDE method proposed by Botev et al. (2010) does not require an optimization procedure to compute the bandwidth parameter, it is fast and suitable for online analyses.

In addition to the proposed kernel derivative, three other methods were also used to compare this study. The first-order difference operator was first used as represented in Equation (2);


(2) $${\text{DF}}_{1}\left({\text{x}}_{i}\right)=\frac{1}{{\text{T}}_{s}}\left({\text{x}}_{i}-{\text{x}}_{i-1}\right)$$

where x_*i*_ is the kernel sample, and T_*s*_ is the sampling time. This formula approximates the continuous-time derivation. However, it amplifies the high-frequency fluctuations if presented in the kernel density output. The point central difference operator (also known as three-point Lagrange derivative) (Marble et al., 1981) was further used. In this method, two consecutive outputs of the first-order difference are averaged using Equation (3):


(3) $${\text{DF}}_{2}\left({\text{x}}_{i}\right)=\frac{1}{2\times {\text{T}}_{s}}\left({\text{x}}_{i}-{\text{x}}_{i-2}\right)$$

The direct KDE proposed by Sasaki et al. (2014) was also used. Assuming that *n* i.i.d. samples $X={\left\{x_{i}\right\}}_{i=1}^{n}$ driven from unknown density *p(x)*, belonging to R_*d*_ are available, the goal is to estimate the *k^th^* order (partial) derivative of *P(x)*.


(4) $${\text{p}}_{k,j}\left({\text{x}}_{i}\right)=\frac{\partial^{k}}{\partial {\text{x}}_{1}^{j_{1}}\partial {\text{x}}_{2}^{j_{2}}\ldots \partial {\text{x}}_{d}^{j_{d}}}$$

where j_*i*_ ∈ {0, 1,…, k}, j_1_ + j_2_ + … + j_*d*_ = *k* and *d* is the dimension of *x*. in a case, when *k* = 2 and *d* = 2, p_*k,j*_(x) corresponds to the Hessian matrix of p(x) as follow:


(5) $$p_{2,j}\left(x\right)=\left[\frac{\partial^{2}}{\partial x_{1}^{2}}\frac{\partial^{2}}{\partial x_{2}\partial x_{1}}\frac{\partial^{2}}{\partial x_{1}\partial x_{2}}\frac{\partial^{2}}{\partial x_{2}^{2}}\right]$$

In this method, the *k^th^* order derivative of density P_*k*_(x) is modeled by the pilot function *g_*k*_(x)*. Then, the pilot function is learned to minimize the mean integrated square error (MISE) criterion, as shown in Equation (6):


(6) $${\text{J}}_{j}\left({\text{g}}_{k,j}\right)=\int {\left\{{\text{g}}_{k,j}\left(x\right)-{\text{p}}_{k,j}\left(x\right)\right\}}^{2}\text{dx}-\int {\left\{{\text{p}}_{k,j}\right\}}^{2}\text{dx}$$

where J_*j*_(g_*k,j*_) is the mean integrated square error, finally, a density derivative estimator g_*k,j*_(x) is obtained by minimizing the expression (5) (Sasaki et al., 2014).

In this study, sEMG samples over predefined windows were used for KDE. The time samples of the sEMG signal in each channel for each repetition of each movement were divided into three equal time windows during the contraction, and their density was computed over the middle window. After this step, three feature sets were computed from the estimated density, as detailed in the following.

#### Trimmed Mean of the density

The central tendency of the density values obtained by KDE was estimated using the trimmed mean operator. Random variables with asymmetric distributions have many applications in non-stationary signal processing. Due to discrepant values in samples, the standard sample mean is not a robust parameter. Various methods have been proposed in the literature to improve the stability of the sample mean (Horn and Kafadar, 2006). A standard method is based on removing the larger or smaller observations than certain limits and then computing the mean of the remaining observations, as in Equation (7) (Burdett, 1996).


(7) $$trimmedmean=E\left\{x|lower_{th}<x<higher_{th}\right\}$$

where lower_*th*_ is the lower limit and higher_*th*_ is the higher limit for the mean calculation. Our study removed 5% of the data in the low range and 5% in the high range.

#### The Entropy of the Density

The complexity of the density values was assessed using the Approximate Entropy (ApEn) (Pincus, 1991). The ApEn determines the conditional probability of similarity between a chosen data segment of a given duration and the next set of segments of the same duration; the higher the probability, the smaller the ApEn value, indicating less complex data. We used ApEn of the estimated density of the sEMG signal. Calculating ApEn requires prior determination of two unknown parameters: *m*, the embedding dimension, and *r*, the tolerance value. These parameters are usually estimated based on an exhaustive search, which is time-consuming. Conversely, in this study, we used the fast heuristic stochastic tuning approach proposed by Chon et al. (2009).

#### Trimmed Mean Absolute Value of the Density Derivative

The density derivative, a versatile tool in statistical data analysis, has been previously used for mean shift clustering in image processing (Duong et al., 2016) and nearest-neighbor Kullback-Leibler (KL) divergence approximation for feature selection (Tangkaratt et al., 2015) in machine learning. A traditional approach is the computation of the derivate of the density values obtained by KDE. Although the direct kernel density derivative estimator (KDDE) proposed by Sasaki et al. (2014) is a suitable choice for the kernel derivation, its computational complexity is high. Therefore, we used a simple estimator for density derivative by computing the gradient of the kernel density estimation (Schuster, 1969; Jordanić et al., 2017), which has less complexity and comparable performance. When the kernel *K* is differentiable *r* times, then the r*^th^* density derivative estimate ${\hat{f}}^{\left(r\right)}\left(x\right)$ of *f* can be computed as Equation (8):


(8) $${\hat{f}}^{\left(r\right)}\left(x\right)=\frac{1}{nh^{r+1}}\sum_{i=1}^{n}K^{\left(r\right)}\left(\frac{x-x_{i}}{h}\right)$$

where K(r) is the r*^th^* derivative of the kernel *K*.

A widely used kernel is the Gaussian kernel with zero mean and unit variance, that is $K\left(u\right)=\frac{1}{2\pi }e^{-\frac{u^{2}}{2}}$. We used the Gaussian kernel so that the entire r*^th^* derivatives could be easily estimated through the r*^th^* derivative of the kernel estimate. A similar algorithm can be derived for other kernels as well. The r*^th^* derivative of the Gaussian kernel K(u) is given by *K*^(*r*)^ (*x*) = (−1)^*r*^ *H*_*r*_(*x*) *K*(*x*), where *H*_*r*_(*x*) is the r*^th^* Hermite polynomial. Hence, from Equation (8), the density derivative estimate with the Gaussian kernel can be written as Equation (9):


(9) $${\hat{f}}^{\left(r\right)}\left(x\right)=\frac{{\left(-1\right)}^{r}}{\sqrt{2\pi }nh^{r+1}}\sum_{i=1}^{n}H_{r}\left(\frac{x-x_{i}}{h}\right)e^{\frac{-{\left(x-x_{i}\right)}^{2}}{2h^{2}}}$$

where *r* indicates the degree of derivation, *h* is the estimated bandwidth of the kernel, *x* is the vector of samples, and *n* is the number of samples.

The trimmed mean absolute value of the kernel derivative was used as a feature. The trimming parameters were the same as for the trimmed mean of the density.

The new sEMG features were compared with classic features as well as wavelet features. The classic features included the Root Mean Square (RMS), the Waveform Length (WL) (Boostani and Moradi, 2003), and the Mean Absolute Value (MAV). The wavelet features were the energies of the Discrete Wavelet Transform (DWT) coefficients of the EMG signals over three scales, using the sym4 as mother wavelet (Boostani and Moradi, 2003).

### Classification

#### Conventional (Sequential) Control

##### Feature Selection for Ensemble Learning

The features were extracted from the middle sEMG epoch for each of the 12 channels. The sEMG signals recorded from nearby channels are usually correlated. Moreover, the recorded data may contain either redundant or irrelevant features and can thus be removed. For this purpose, we used a filter-based feature selection method, Correlation-based feature selection (CFS), which uses a heuristic based on correlation to evaluate the importance of the features (Hall, 2000). This method hypothesizes that good feature subsets contain highly correlated features with the class yet uncorrelated with each other. Based on this hypothesis, the heuristic is formalized as follow:


(10) $${\text{Merit}}_{s}=\frac{\text{k}\underline{{\text{r}}_{c-f}}}{\sqrt{\text{k}+\text{k}\left(\text{k}-1\right)\underline{{\text{r}}_{f-f}}}}$$

where Merits is the heuristic “merit” of a feature subset S containing k features, $\underline{r_{c-f}}$ the average feature-class correlation, and $\underline{r_{f-f}}$ the average feature-feature inter-correlation. In order to find the best subset, CFS uses a heuristic search known as Best First (Rich and Knight, 1991) to evaluate its usefulness. CFS thus first calculates a matrix of feature-class and feature-feature correlations from the data and then searches the feature subset space using the best first search. The best first search starts with an empty set of features and generates all possible single feature expansions. The subset with the highest evaluation is chosen and expanded in the same manner by adding single features. If expanding subset results in no improvement, the search drops back to the following best-unexpanded subset and continues from there. The best subset found is returned when the search terminates. CFS uses a stopping criterion of five consecutive fully expanded non-improving subsets (Hall, 2000). CFS usually outperforms the wrapper methods on small datasets, such as our sEMG dataset, based on accuracy and efficiency.

##### Classification and Ensemble Learning

As the focus of this study is to introduce new features, commonly used classifiers were applied as a base learner for Ensemble learning. These were Linear Discriminant Analysis (LDA), Support Vector Machine (SVM), and Multi-Layer Perceptron (MLP). An ensemble of classifiers is a set of classifiers whose individual decisions are combined to obtain a system to outperform each of its members. The ensemble members, known as base learners or base classifiers, must be both accurate and diverse to achieve this goal. A classifier is accurate if its classification error is lower than obtained when the classes are assigned randomly. Two classifiers are diverse if they make errors on different instances.

In our analysis, an MLP with one hidden layer was trained with the Back Propagation algorithm. The number of neurons in the hidden layer was set to 15 based on trial and error, and its activation function was the tangent sigmoid function for the hidden layer and linear function for the output layer.

Non-linear SVM was applied with the radial basis function (RBF) kernel. The soft-margin parameter and the radius of the RBF kernel were set with the method proposed by Wu and Wang (2009).

For each classifier, the class output, and the posterior probability matrix, the degree of decision making given to each class, were derived. The Behavioral Knowledge Space (BKS) method was used for combining the output of the base learners [for review see Polikar (2006)].

#### Simultaneous Control

To enhance the performance of myoelectric prostheses, it is essential to control multiple tasks simultaneously. For this reason, the features proposed in this study were used to classify simultaneous movements. This section used the database registered by Ameri motion (Ameri et al., 2018).

##### Classification

The XGBoost (eXtreme Gradient Boosting) algorithm was used to classify simultaneous movements. The XGBoost algorithm is an implementation of the decision tree gradient boosting designed for high speed and performance. Due to the fast learning and efficient memory, this algorithm was chosen. This algorithm combines several weak predictors to create a robust classifier (Swamynathan, 2017; Nguyen et al., 2020).

Parameters of the XGBoost were selected by testing the accuracy under different estimator numbers, maximum tree depths, and learning rate. The number of boosted trees was set to 600 (estimators). Furthermore, the maximum tree depth and learning rate were set to 18 and 0.1, respectively.

#### Performance Evaluation

The performance of the proposed classification system was assessed in terms of accuracy and the number of subjects with at least one wholly misclassified movement. For each movement class C_*i*_ (i ϵ{1,…, c}; c = the number of classes), the following parameters were extracted based on the traditional signal detection theory (Sokolova and Lapalme, 2009):

True Positive (TP_*i*_): the number of samples correctly identified as class C_*i*_;
True Negative (TN_*i*_): the number of samples correctly identified as any class except the class C_*i*_;
False Positive (FP_*i*_); the number of samples incorrectly identified as class C_*i*_;
False Negative (FN_*i*_); the number of samples belong to class C_*i*_ but incorrectly assigned to other classes;

(11) $${\text{precision}}_{i}\left({\text{PR}}_{i}\right)=\frac{{\text{TP}}_{i}}{{\text{TP}}_{i}+{\text{FP}}_{i}}$$

(12) $${\text{Recall}}_{i}\left({\text{RL}}_{i}\right)=\frac{{\text{TP}}_{i}}{{\text{TP}}_{i}+{\text{FN}}_{i}}$$

(13) $${\text{Accuracy}}_{i}\left({\text{Acc}}_{i}\right)=\frac{{\text{TP}}_{i}+{\text{TN}}_{i}}{{\text{TP}}_{i}+{\text{FP}}_{i}+{\text{TN}}_{i}+{\text{FN}}_{i}}$$

(14) $$\text{F}-{\text{score}}_{i}\left({\text{F}}_{i}\right)=\frac{2\text{P}{\text{R}}_{i}\times {\text{RL}}_{i}}{{\text{PR}}_{i}+{\text{RL}}_{i}}$$

For each subject, the overall validity of the proposed multi-class classification system was assessed using the micro (F_*micro*_) and macro (F_*macro*_) averaged F-scores and the overall accuracy. The macro averaged F-score is the average of the entire F-scores calculated for the entire classes (Equation 14) while, the micro averaged F-score is assessed by calculating the F-score over the micro averaged Precision (PR_*u*_) and Recall (RL_*u*_) (Equations 15, 16; Sokolova and Lapalme, 2009). The overall accuracy was then calculated as the sum of the diagonal elements of the multi-class confusion matrix divided by the sum of the entire confusion matrix elements. It is easily shown that F_*micro*_, PR_*u*_, RL_*u*_, and the overall accuracy measure are identical.


(15) $$F_{macro}=\frac{\sum_{i=1}^{c}F_{i}}{c}$$

(16) $$PR_{u}=\frac{\sum_{i=1}^{c}TP_{i}}{\sum_{i=1}^{c}\left(TP_{i}+FP_{i}\right)}$$

(17) $$RL_{u}=\frac{\sum_{i=1}^{c}TP_{i}}{\sum_{i=1}^{c}\left(TP_{i}+FN_{i}\right)}$$

Theoretically, when at least one of the movements is completely misclassified in a fold (i.e., TP = 0 in the entire two recording repetitions), the macro averaged F-score (F_*macro*_) could not be calculated (shown as NaN in the results). The number of subjects with at least one misclassified movement was then determined based on this measure. In these cases, the number of correctly classified movements was reported for each subject.

Repeated hold out (*N* = 3) validation method was used to assess the classifier’s performance in the conventional myoelectric control. The repeated method was used in order to overcome a possible pessimistically biased error estimate. For each subject, the data were split into two mutually exclusive training (four repetitions data) and test sets (two repetitions data), that is twofolds (Gijsberts et al., 2014). Such validation was repeated three times (Split1, Split2, Split3) by changing the permutation of the recorded data repetitions as below (Gijsberts et al., 2014):

Split1: test set = {Rep_1_, Rep_4_}, training set = {Rep_2_, Rep_3_, Rep_5_, Rep_6_}.
Split2: test set = {Rep_2_, Rep_5_}, training set = {Rep_1_, Rep_3_, Rep_4_, Rep_6_}.
Split3: test set = {Rep_3_, Rep_6_}, training set = {Rep_1_, Rep_2_, Rep_4_, Rep5}.

Where Rep_*i*_ represents the i*^th^* repetition of each movement. The results of different folds were then averaged for each movement.

For Ameri’s dataset, a fourfolds cross-validation method was used. The data was divided equally into fourfolds, threefolds were used for training while the remaining fold was used for evaluation, and this procedure continued until the entire folds were tested.

### Statistical Analysis

All statistical analyses and calculations were performed using the SPSS statistical package, version 18.0 (SPSS Inc., Chicago, IL, United States). Data are reported as means ± standard deviation. The normality of the data was checked using the Kolmogorov–Smirnov test. The Generalized Linear Model (GLM) was used for assessing the correlation between the proposed features and the movement classes. The association between the movement classification and the remaining forearm was assessed using the partial correlation, controlling for the DASH (Disability of the Arm, Shoulder and Hand) score (Atzori et al., 2014a). We used Generalized Estimating Equation (GEE) method (Hardin and Hilbe, 2014) for modeling factors associated with repeated responses [i.e., overall accuracy of the proposed classification system in different movement class categories (Figure 1)]. A Kruskal–Wallis (KW) one-way analysis of variance was used to test for differences in the overall accuracy of the LDA classifier using trimmed mean absolute value of derivative density (TMAVDD) feature calculated using different kernel derivative estimation methods. When KW identified a significant difference, the Mann–Whitney *U*-test with Bonferroni correction was used for pair-wise comparisons. Fleiss’ kappa (Gwet, 2014), a statistical measure for assessing the reliability of agreement between the output of the ensemble classification system and that of the gold standard, was calculated on the pooled test-set confusion matrices on the analyzed threefolds in the intact and amputee subjects. McNemar’s test (MN) was used to compare different classifiers to identify whether one classifier statistically significantly outperforms the other (Dietterich, 1998). A two-sided *P*-value of < 0.05 was considered statistically significant. Finally, the proposed movement detection system was implemented using Matlab and Statistics Toolbox Release 2011a (The MathWorks, Inc., Natick, Massachusetts, United States).

## Results

### Ninapro Database

The features trimmed mean of density (TMD), TMAVDD, and entropy of density (ED) were extracted from each channel to obtain a feature vector in 36 dimensions. The number of features was reduced using the CFS method ranging from 17 to 29. Such features were then randomly distributed to the base learner classifiers. The performance of the proposed ensemble classification system for each subject is shown in Supplementary Tables 1, 2 for intact and amputee subjects, respectively.

For intact subjects (dataset 2), overall, in the training set, the average (micro) F-Score, and (macro) F-Score across the full subject sample were 99.96 ± 0.19, and 99.99 ± 0.01 in percent, respectively. These values for the test set were 98.32 ± 1.93, and 98.23 ± 2.02 in percent, respectively.

For amputees (dataset 3), in the training set, the average (micro) Fscore, and (macro) Fscore across the entire subject sample were 97.65 ± 3.2, and 99.88 ± 0.16 in percent. Such values for the test set were 90.25 ± 10.5 and 99.5 ± 0.53 in percent, respectively. The partial correlation between (micro) Fscore and the remaining forearm (%) was 0.752 (*P*-value = 0.020).

Fleiss’ kappa (inter-rater reliability) for the movement detection in intact and amputee subjects were 0.9660 [C.I. 95%: (0.9659–0.9661)] and 0.8123 [C.I. 95%: (0.8119–0.8126)], respectively (*P*-value < 0.001). It thus showed “almost perfect agreement” between the output of the ensemble classifier and that of the gold standard, indicating the observed agreement was not accidental in the entire subjects (Fleiss et al., 2013).

The performance of the proposed classification system was further assessed based on the total misclassification for a specific movement for the entire subject sample, sets (training and test), and six repetitions (Table 1). In fact, instead of analyzing each fold, including two recording repetitions, each repetition was analyzed. Overall, the average misclassified movements, and their percentage in the entire recordings were 1.40 ± 1.38 and 1.75 ± 1.73 (%), respectively, in the entire recording sets and subjects.

**TABLE 1:** The number of misclassifications for each movement in each hold out repetition scenario.

Over intact subjects																					
**# mov.**	**1**	**2**	**3**	**4**	**5**	**6**	**7**	**8**	**9**	**10**	**11**	**12**	**13**	**14**	**15**	**16**	**17**	**18**	**19**	**20**	**Sum**
Split1	1	1	1	1	1	0	0	2	2	1	3	2	1	1	2	0	0	5	5	5	34
Split2	1	1	2	0	0	2	0	0	1	0	1	1	2	0	0	1	1	1	3	0	17
Split3	1	0	1	0	1	0	0	1	0	1	0	0	2	0	1	1	1	3	0	2	15

**# mov.**	**21**	**22**	**23**	**24**	**25**	**26**	**27**	**28**	**29**	**30**	**31**	**32**	**33**	**34**	**35**	**36**	**37**	**37**	**39**	**40**	**Sum**

Split1	3	2	2	2	1	1	3	5	0	7	4	2	3	1	2	2	0	0	1	0	41
Split2	5	1	2	1	1	2	1	1	1	4	3	2	1	0	0	1	1	1	1	0	29
Split3	2	1	5	0	1	1	3	3	3	3	2	1	0	0	2	1	2	0	1	1	32

**Over amputee subjects**																					

**# mov.**	**1**	**2**	**3**	**4**	**5**	**6**	**7**	**8**	**9**	**10**	**11**	**12**	**13**	**14**	**15**	**16**	**17**	**18**	**19**	**20**	**Sum**

Split1	3	4	3	1	2	2	3	4	3	0	3	1	5	2	6	5	5	6	1	5	64
Split2	2	1	2	1	3	3	2	1	3	0	3	2	6	3	3	2	8	8	4	4	61
Split3	1	2	1	1	0	1	4	3	5	1	4	1	5	3	2	1	4	6	2	4	51

**# mov.**	**21**	**22**	**23**	**24**	**25**	**26**	**27**	**28**	**29**	**30**	**31**	**32**	**33**	**34**	**35**	**36**	**37**	**37**	**39**	**40**	**Sum**

Split1	3	6	5	4	5	2	4	3	3	5	4	9	5	5	4	0	5	0	2	1	75
Split2	5	6	4	4	4	4	4	8	7	8	4	9	3	5	8	0	6	1	1	0	91
Split3	6	4	3	2	5	3	1	3	8	8	3	3	5	6	7	1	2	0	0	1	71

*#mov. (movement number) for each considered split; the number of misclassifications was calculated as the total number of cases in the entire training or test sets and 40 subjects, in which the specific movement was not correctly (either False Positive or False Negative error) estimated.*

Eighty percent of the movements had less than four misclassified cases in the entire validation folds and subjects for intact subjects. The overall performance of the proposed algorithm for the class categories (b: movement classes 9–17) and (c: classes 18–40) was similar but was statistically significantly lower than that of the category (a: classes 1–8) (GEE; *P*-value < 0.05). For amputee subjects, 83% of the movements had less than seven misclassified cases in the entire validation folds and subjects. The overall performance of the proposed algorithm for the class categories (b: movement classes 9–17) and (c: classes 18–40) was similar but was statistically significantly lower than that of the category (a: classes 1–8) (GEE; *P*-value < 0.05).

The performance of the proposed system was compared with that of other commonly used methods and features in terms of the number of subjects with at least one completely misclassified movement in a validation fold (Table 2). DWT features were calculated using the Symlet mother wavelet with three vanishing moments up to the third level decomposition (Boostani and Moradi, 2003). DWT (with LDA classifier) showed better performance (11 misclassifications).

**TABLE 2:** The comparison between different methods in terms of the number of subjects with at least one completely misclassified movement in a validation fold in intact subjects.

	Commonly used features												New proposed features									
Features	WL			MAV			RMS			DWT			TMD			TMAVDD			ED		All new features	
Classifiers	LDA	SVM	MLP	LDA	SVM	MLP	LDA	SVM	MLP	LDA	SVM	MLP	LDA	SVM	MLP	LDA	SVM	MLP	LDA	SVM	MLP	Ensemble of learners
Whole period	20	38	40	14	38	40	29	38	40	11	17	40	15	29	40	40	40	40	40	40	40	4
Middle part	40	40	40	40	40	40	40	40	40	40	40	40	**4**	18	40	40	40	40	40	40	40	**0**

*WL, waveform length; MAV, Mean Absolute Value; RMS, Root Mean Square; DWT, Discrete Wavelet Transform; TMD, Trimmed Mean of Density; TMAVDD, trimmed mean absolute value of derivative density; ED, Entropy of density. Bold values indicate that the best methods for analyzing the middle part.*

Supplementary Figure 1 shows the number of ideally classified movements in each intact subject’s best and worst folds. For the intact subjects, at least 28 movements were ideally classified, while for the amputees, excluding subject six, a number of 15 movements were ideally classified in the entire analysis folds. Moreover, the performance of the proposed classification system was assessed on each movement of the entire intact subjects (Supplementary Figure 2). A similar plot was provided for the amputees (Supplementary Figure 3).

**FIGURE 2:**
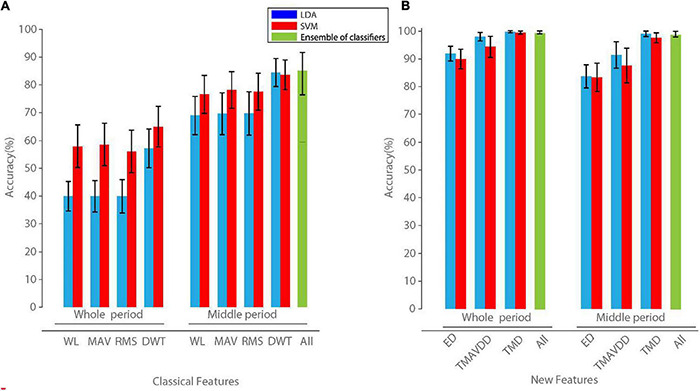
The overall classification accuracy of three new features individually as well as their combination **(A)**, and classic features **(B)** in the whole and also middle period of recording in the intact subjects. Individual features were classified using linear discriminant analysis (LDA) and support vector machine (SVM), and ensemble of LDA, SVM, and multi-layer perceptron (MLP) classifiers. The results of the MLP classifier were not shown as they were very low, compared to those of LDA and SVM. Error bars show standard deviation over the test folds. WL, waveform length; MAV, mean absolute value; RMS, root mean square; DWT, discrete wavelet transform; ED, Entropy of density; TMD, Trimmed Mean of Density; TMAVDD, trimmed mean absolute value of derivative density.

The effect of ensemble learning to improve the performance of the proposed classification system was shown in Figure 2 over intact subjects. The three new features ED, TMAVDD, and TMD were first separately used with the LDA and SVM classifiers in this figure. LDA with TMD showed the best performance with average accuracy (98.99 ± 1.36). Ensemble learning not only improved the average accuracy (99.91 ± 0.10), but it also reduced the number of cases with at least one completely misclassified movement in a validation fold from 4 to 0 (Table 2).

**FIGURE 3:**
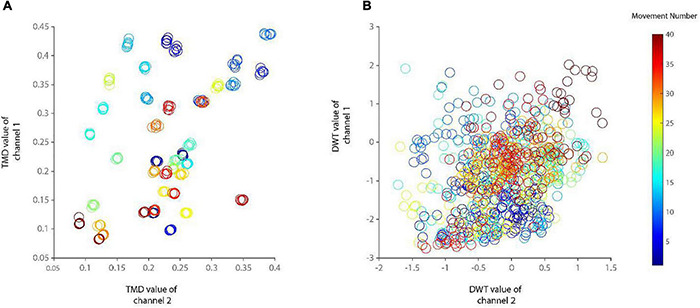
The scatter plot of the features trimmed mean of density (TMD) **(A)** and discrete wavelet transform (DWT) **(B)** in the second versus first recording channels in a subject. Features related to a specific movement calculated at different recording data repetitions were grouped on the left panel. The color bar shows the colors assigned to each recording channel.

The class discrimination capability of the first proposed feature (TMD) was shown compared to that of DWT in Figure 3. The proposed movement detection algorithm showed an overall accuracy of 99.91 ± 0.10 across the entire subject sample and movements. No movement was missed entirely in a validation fold that includes two recording data repetitions (Table 2). Each analyzed fold was further examined, and the average misclassified movements in the entire recordings were 1.40 ± 1.38 for intact subjects. The success of the proposed algorithm was highly dependent on the extracted features and ensemble learning.

The reduction in the feature vector in each recording electrode compared to that of commonly used features resulted in higher accuracy (Figure 2) and reduced the number of misclassified movements (Tables 1, 2). Meanwhile, the ensemble learning used in our study further improved the number of misclassified movements (Table 1) and the overall accuracy (Figure 2).

**FIGURE 4:**
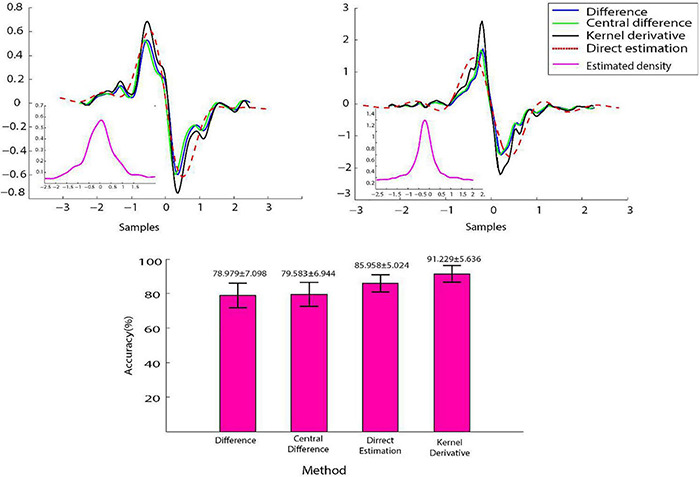
The effect of different methods of estimating the kernel density derivative on the overall performance of classification. Upper panel represents the kernel density and its derivatives estimated using four methods (basic difference, central difference, kernel derivative, and direct kernel derivative) on a signal for the movements no. Twenty (left) and 13 (right). Lower panel shoes the overall classification performance of the linear discriminant analysis (LDA) classifier with trimmed mean absolute value of derivative density (TMAVDD) feature when derivative of estimated density was calculated using those four methods.

Different KDDE methods were used to estimate the kernel density derivative. LDA classifier was then used with the feature TMAVDD calculated for each recording channel, and the overall performance was reported for the entire subjects and movements (Figure 4). The performance of the proposed kernel derivative estimation was statistically significantly better than the three other methods (KW; *P*-value < 0.05).

We also implemented an online version of the proposed movement detection algorithm. TMD features with LDA classifier were used to analyze epochs of 300, 200, 100, and 50 ms and were validated on three split sets. The average accuracy of 40 hand-movement classifications in the entire intact subjects on the test sets was reported. The average running time of the entire classification system (feature extraction, feature selection, and classification) was also reported on the training and test sets (Table 3). The analysis was performed on an Intel i5-4300U 1.90 GHz CPU with 8 GB of RAM. Table 3 indicates that, for example, the online movement detection method could be used for the analysis window of 200 ms with an average accuracy of 82% and a processing time of 63 ms.

**TABLE 3:** The performance of the online classification system on different epoch widths over the entire movements and intact subjects as well as its average running time on the training and test sets.

Epoch width (ms)	Training time (ms)	Test time (ms)	Overall accuracy
300	67.72 ± 7.98	67.69 ± 7.96	85.75 ± 6.98
200	63.44 ± 6.62	63.43 ± 6.62	82.20 ± 7.72
100	56.25 ± 5.64	56.24 ± 5.64	73.97 ± 9.04
50	53.53 ± 5.60	53.53 ± 5.60	61.51 ± 8.99

*The average running time included the feature extraction, selection and classification times. The overall accuracy was calculated on three tests sets in the Split1, Split2, and Split3 sets.*

### Ameri’s Database

**FIGURE 5:**
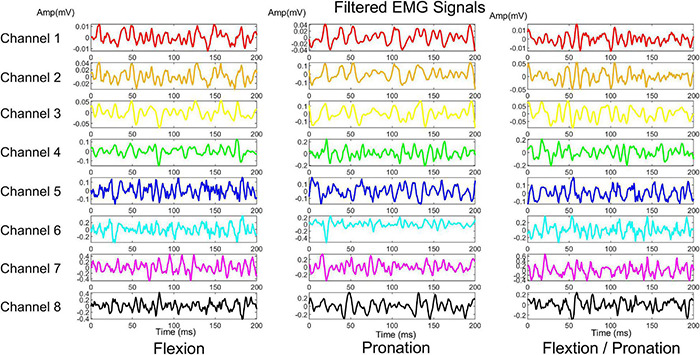
A sample filtered signal array for three movements of Ameri’s dataset.

Epochs of 200 ms filtered sEMG signals, during flexion, pronation, and simultaneous flexion, and pronation were shown in Figure 5. The corresponding raw sEMG signals were shown in Supplementary Figure 4. In the Ameri dataset (Ameri et al., 2018), the average classification accuracy, F-Score (micro), and F-Score (macro) (mean ± standard deviation) for all subjects for the XGBoost classifier were 99.71 ± 0.08 and 98.79 ± 0.37, respectively. The performance of the algorithm for each subject was shown in Supplementary Table 3. Moreover, the cross-validated confusion matrix and the target versus predicted classes were provided as the following for each subject: https://doi.org/10.6084/m9.figshare.18865121.

**FIGURE 6:**
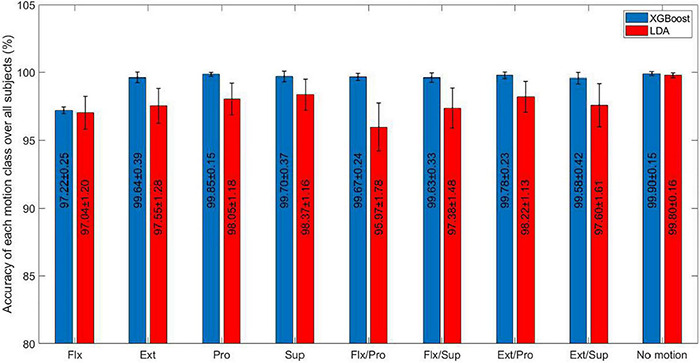
The comparison between the performance of the eXtreme gradient boosting (XGBoost) and linear discriminant analysis (LDA) classifiers for each movement class. Error bars show standard deviation over the test folds. Flx, Flexion; Ext, Extension; Pro, Pronation; Sup, Supination.

The performance of the XGBoost was compared with that of the LDA, as one of the most commonly used classifiers applied to movement detection (Supplementary Table 3). In both classifiers, the proposed new features were used. The average classification accuracy, F-Score (micro), and F-Score (macro) (mean ± standard deviation) for all subjects for the LDA classifier were 97.85 ± 0.10 and 91.02 ± 0.37 in percent, respectively. The XGBoost significantly outperformed the LDA classifier (MN; *P*-value < 0.05). Moreover, the performance of the XGBoost and LDA classifiers was compared for each movement class (Figure 6).

## Discussion

### Comparison With the State-of-the-Art

Gijsberts and Caputo (2013) used the combination of DWT, RMS, and HIST sEMG features with Kernel Regularized Least Squares (KRLS) classifier with the exp-χ2 kernel to discriminate 40 hand and wrist movements in 20 intact subjects (Ninapro V.2 dataset). The authors reported an overall accuracy of 77.75%. The accelerometer information was further added to the DWT features to reach an accuracy of 82.59%. Gijsberts et al. (2014) used DWT, RMS, and HIST features with KRLS classifier to discriminate 40 hand and wrist movements (and the rest posture) in 40 intact subjects (Ninapro V.2 dataset). The authors reported an overall accuracy of 77.48%. The accelerometer information was further added to the DWT feature, and the accuracy of 82.49% was obtained. In a recent study, Li et al. (2017) proposed the combination of sEMG and electroencephalography (EEG) to improve the accuracy of hand movement detection. They used four time-domain features with an LDA classifier to discriminate five hand motion classes in four transhumeral amputees. The authors reported overall accuracy (per five movements per four subjects) of 91.7%.

We used TMD, TMAVDD, and ED sEMG features with the LDA classifier to discriminate 40 hand and wrist movements in 40 intact subjects (Ninapro dataset). The correlation-based feature selection was used to reduce the feature space. The overall accuracy was 98.99, 91.23, and 83.00%, for such features, respectively (Figure 2). When those three features were used in ensemble learning using LDA, SVM, and MLP base classifiers, the overall accuracy increased to 99.91%. Atzori et al. tested Convolutional Neural Network for classifying 40 movements on both NinaPro datasets 2 and 3. The classification accuracy obtained with convolutional neural networks using their proposed architecture was 60.27 ± 7.7% on dataset 2 and 38.09 ± 14.29% on amputees (dataset 3) (Atzori et al., 2016).

Geng et al. (2016) proposed a real-time method for recognizing eight hand gestures. Their method was based on HDsEMG image in which instantaneous values of recorded sEMG via an array of the electrode are mapped to an image and classified using a deep convolutional neural network. They also evaluated the method on the NinaPro dataset 2. They achieved recognition accuracy of 76.1% over DB2. AbdelMaseeh et al. (2016) extracted multi-channel EMG activation trajectories and classified the extracted trajectories using a metric based on multi-dimensional dynamic time warping. An accuracy of 89% with an average movement error rate of 0.09 was obtained. The best results reported on the NinaPro dataset 3 were by Atzori et al. (2014b), who used both sEMG and accelerometry data. They showed a maximum average classification accuracy for 40 movements in five amputees of 61.14%. These results showed that our method based on Kernel Density of sEMG signal combined with an ensemble of learners outperforms state-of-the-art methods for movement recognition, even for the sEMG signals from amputee subjects.

In our study, the repeated hold-out validation (*N* = 3) was used to overcome a possible pessimistically biased error estimate and guard against testing hypotheses suggested by the data (Type III errors). In each hold-out, four data recording repetitions were used for training and two for testing. The second hold-out was the same as what was used in Gijsberts et al. (2014) for validation.

Ameri et al. (2018) used four time-domain features with CNN and SVM classifiers to discriminate nine simultaneous movements in 17 intact subjects (Ameri dataset). The authors reported overall classification accuracies for the CNN and SVM, 91.61 ± 0.39, and 90.63 ± 0.31, respectively.

We used TMD, TMAVDD, and ED sEMG features with the XGBoost and LDA classifier to discriminate nine simultaneous movements in 17 intact subjects (Ameri dataset). The overall accuracy for the XGBoost and LDA were 99.71 ± 0.08 and 97.85 ± 0.10%, respectively.

Thus, the comparison of our result with the state-of-the-art showed that the proposed features, regardless of the type of classifier, can improve movement detection performance. Such features could capture the distribution of the sEMG time samples and their derivatives in a non-parametric framework, and also the complexity of the signal. Moreover, each signal epoch is represented by three features. Lower dimension of the proposed feature vector can generally improve the classification, compared with DWT and the histogram.

### The Formulation of the Proposed Features

The dispersion of the first feature TMD was much lower than that of the two other features. This feature also showed better class separation than that of the other features. Using the TMD feature, the overall classification was better than a univariate application of the other features (Figure 2). The TMAVDD and ED features also showed class separation but were less discriminative than TMD.

The proposed TMAVDD feature uses the same Irregularity Coefficient (dc) concept proposed by Zalewska and Hausmanowa-Petrusewicz (1995). It is defined as Equation (18):


(18) $$d_{c}=\frac{1}{max\left(y_{i};i=1,\ldots ,n_{s}\right)}\sum_{i=1}^{n_{s}-1}\left|y_{i}-y_{i-1}\right|$$

where y_*i*_ are the samples and n_*s*_ is the number of samples. The authors used this feature for decomposing iEMG signals. Such a feature showed an acceptable performance in discriminating different motor unit action potentials (Zalewska and Hausmanowa-Petrusewicz, 1995). If we use the estimated kernel density values as the input of this feature, the nominator of its formula will be the sum of the kernel density derivative samples. However, we divide this by the number of samples and use its trimmed average while the authors normalized it by the peak-to-peak of the input waveform. However, due to its similarity with the first-order difference operator (Equation 1), kernel derivative was shown to be more accurate (Figure 4). TMAVDD is, in fact, a shape measure of estimated kernel density values.

On the other hand, the approximate entropy was used to quantify the amount of fluctuations and the complexity in the time-series data in EMG signals in the literature (Guo et al., 2016). We calculated the complexity of the estimated kernel density values using ApEn. Given that enough information is needed to calculate the entropy (Pincus, 1991), the number of samples could not be reduced for more efficient implementation. Meanwhile, TMD and ED were moderately corrected in our dataset (Spearman’s rho = 0.589; *P*-value < 0.05). Thus, part of the KDE complexity could be assessed by TMD feature, as well. It could be the justification that using TMD feature with LDA classifier resulted in an acceptable overall performance in our analysis (Figure 2).

### Final Considerations

Overall, the misclassified movement percentage was about 2% in our study in the entire intact and amputee subjects. Moreover, at least 28 and 15 movements were ideally classified for the intact and amputee subjects, respectively in the entire analysis folds. The main goal of developing such algorithms is to predict the intent of an amputee to control dexterous, self-powered hand prostheses. Unlike previous studies, we used repeated hold-out analysis (*N* = 3), guarding against testing hypotheses suggested by the data (type III errors). If a movement was not correctly classified in a fold, it was marked as missed.

The application of the proposed system in prosthesis control implies efficient online implementation of the algorithms. The following methods could be expanded for online implementation. We proposed using TMD features and an LDA classifier on 200 ms analysis epochs (Table 3). The average running time of the proposed online algorithm could be further reduced by using the Vectorization package in Microsoft Visual C++ to implement vector and matrix operations efficiently. Such implementation showed a significant running time improvement in the literature (Marateb and Mcgill, 2009).

## Conclusion

In conclusion, we developed a new movement detection algorithm using the sEMG signal recorded from intact subjects and amputees. This classification system is a promising new tool for online prosthesis control. Due to the importance of simultaneous control, the performance of the proposed features was also evaluated for simultaneous movements. The results showed superior performance of these features with respect to previous approaches.

## Data Availability Statement

Publicly available datasets were analyzed in this study. The datasets analyzed for this study can be found in the Ninaweb (http://ninapro.hevs.ch/node/7) and DANS (https://easy.dans.knaw.nl/ui/datasets/id/easy-dataset:109368).

## Ethics Statement

The studies involving human participants were reviewed and approved by Ethics Commission of the Canton Valais (Switzerland) (Ninapro dataset), Shahid Beheshti Medical University (Ameri’s dataset). The patients/participants provided their written informed consent to participate in this study.

## Author Contributions

PG, MN, MJ, HM, MM, and DF participated in the conceptualization, investigation, methodology, and interpretation of the results. PG, MN, and HM participated in the visualization, software, validation, writing—original draft. HM, MM, and DF participated in the project administration and supervision. PG, MN, HM, MM, and DF participated in the formal analysis. MM participated in funding acquisition. MJ, MM, and DF participated in writing—review and editing. All authors read and approved the final manuscript and agreed to be accountable for all aspects of the work.

## Conflict of Interest

The authors declare that the research was conducted in the absence of any commercial or financial relationships that could be construed as a potential conflict of interest.

## Publisher’s Note

All claims expressed in this article are solely those of the authors and do not necessarily represent those of their affiliated organizations, or those of the publisher, the editors and the reviewers. Any product that may be evaluated in this article, or claim that may be made by its manufacturer, is not guaranteed or endorsed by the publisher.
