# Supplementary material for: Kernel Density Estimation of Electromyographic Signals and Ensemble Learning for Highly Accurate Classification of a Large Set of Hand/Wrist Motions
Source: Front Neurosci. 2022 Mar 9;16:796711. doi: 10.3389/fnins.2022.796711 (PMC8959430; doi:10.3389/fnins.2022.796711)
Supplement: Supplementary file 1 [file Data_Sheet_1.PDF]

Supplementary Material

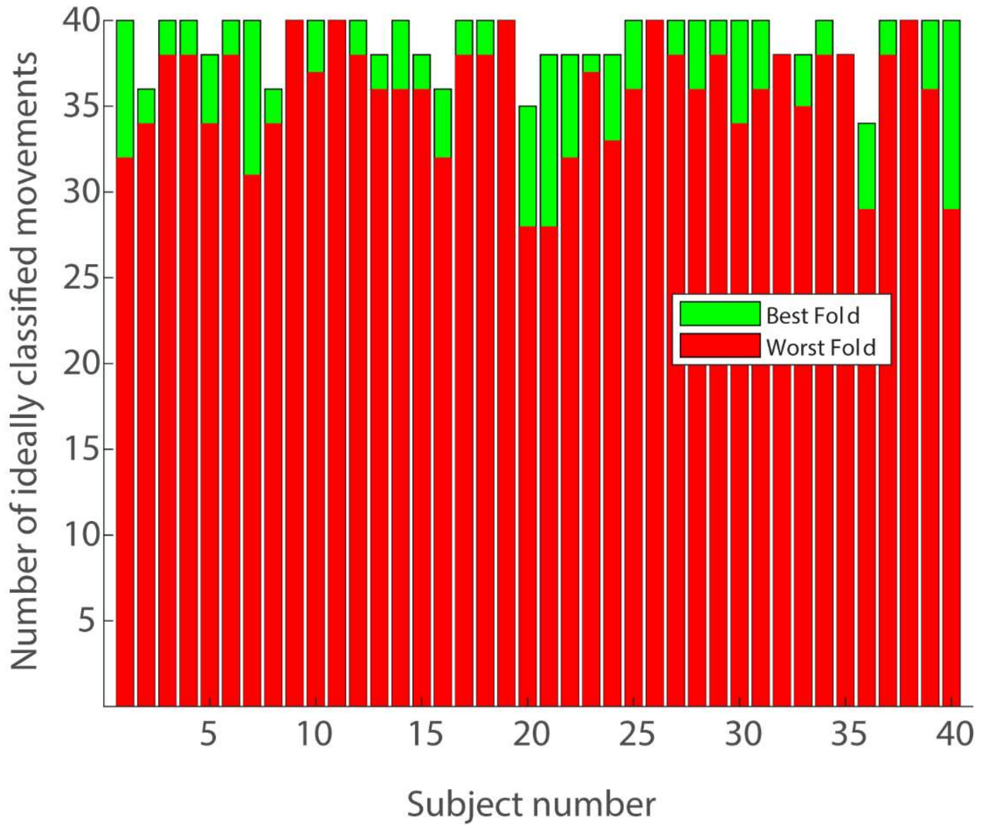

Supplementary Figure 1. Number of ideally classified movements per each subject for best and worst scenarios in the three analyzed folds.

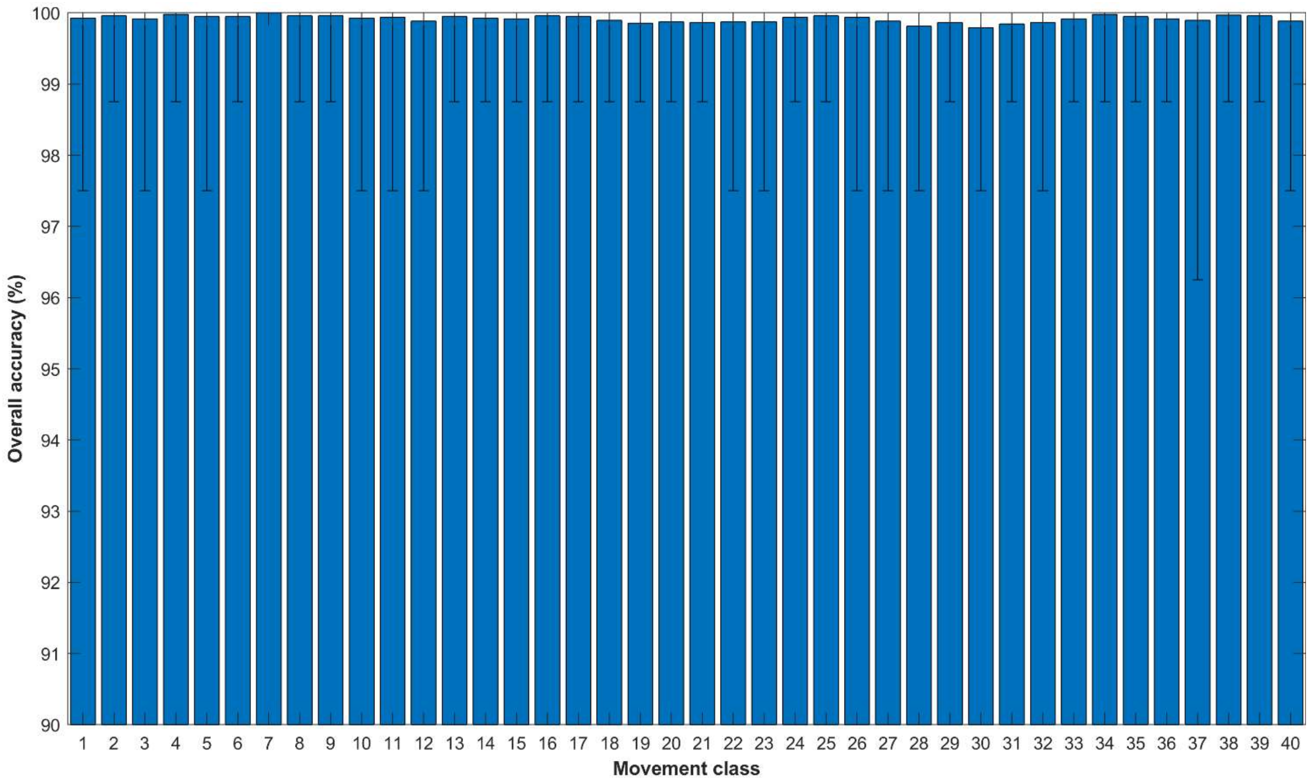

Supplementary Figure 2. The performance of the proposed classification system for each movement of the entire intact subjects. The error bar shows the minimum and maximum classification accuracy on the test folds.

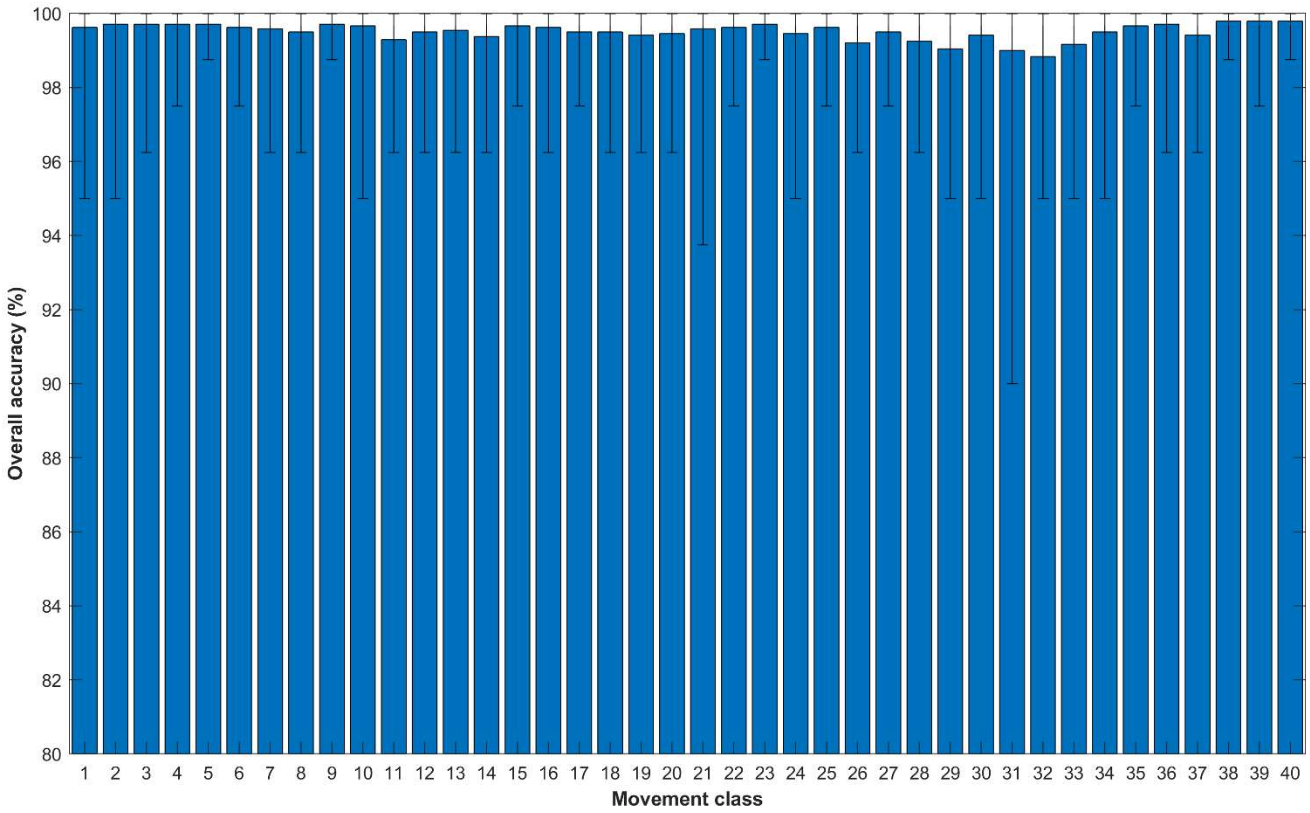

Supplementary Figure 3. The performance of the proposed classification system for each movement of the entire amputees. The error bar shows the minimum and maximum classification accuracy on the test folds.

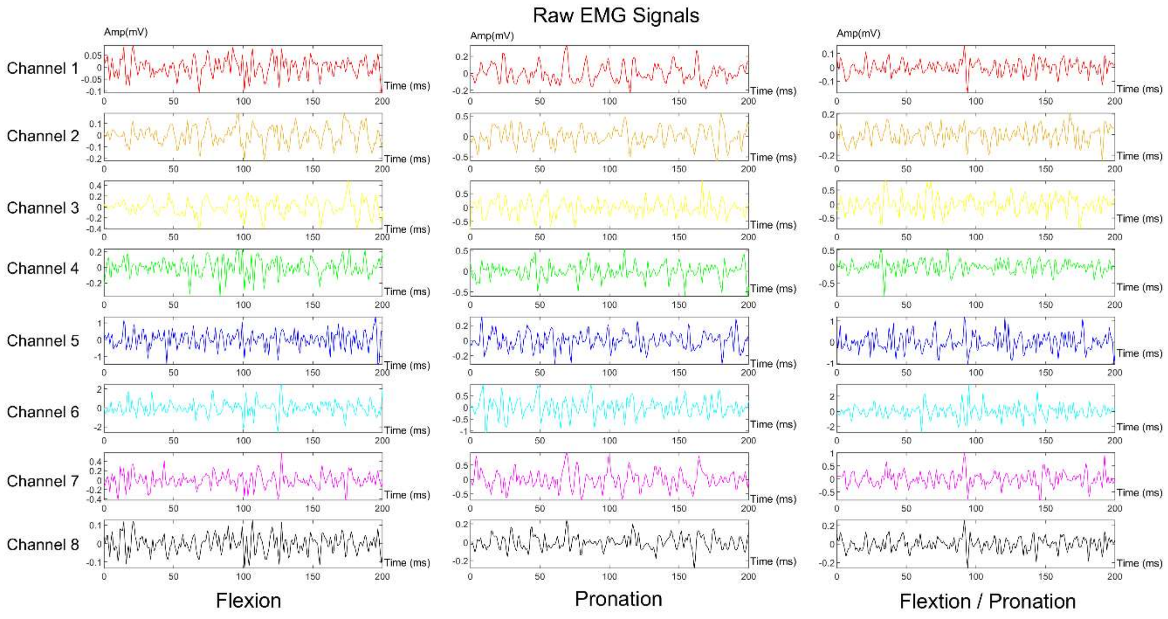

Supplementary Figure 4. A sample raw signal array for three movements of Ameri's dataset.

Supplementary Table 1. The performance of the proposed ensemble classification system for each intact subject for the detection of 40 movements; shown in mean±sd of the 40 movements by 3 analyzed folds.

**Subject number**	**(micro) F-Score**	**(macro) F-Score**	**(macro) Precision**	**(macro) Recall**
1	98.3±2.9	98.2±3.1	98.9±1.9	98.3±2.9
2	96.7±0.7	96.4±0.8	97.8±0.5	96.7±0.7
3	99.2±0.7	99.1±0.8	99.4±0.5	99.±0.7
4	99.6±0.7	99.6±0.8	99.7±0.5	99.6±0.7
5	96.7±1.9	96.5±2.0	97.5±1.7	96.7±1.9
6	99.2±0.7	99.1±0.8	99.4±0.5	99.2±0.7
7	96.7±3.1	96.5±3.3	97.6±2.3	96.7±3.1
8	96.7±1.4	96.6±1.3	98.1±0.4	96.7±1.4

9	100.0	100.0	100.0	100.0
10	99.2±1.4	99.1±1.5	99.3±1.2	99.2±1.4
11	100.0	100.0	100.0	100.0
12	99.6±0.7	99.6±0.8	99.7±0.5	99.6±0.7
13	98.3±0.7	98.2±0.8	98.9±0.5	98.3±0.7
14	98.8±1.3	98.7±1.3	99.2±0.8	98.8±1.3
15	98.3±0.7	98.2±0.8	98.9±0.5	98.3±0.7
16	96.3±1.3	96.0±1.3	97.4±0.9	96.3±1.3
17	99.6±0.7	99.6±0.8	99.7±0.5	99.6±0.7
18	99.6±0.7	99.6±0.8	99.7±0.5	99.6±0.7
19	100.0	100.0	100.0	100.0
20	94.2±2.6	94.0±2.7	96.0±1.6	94.2±2.6
21	96.7±3.6	96.4±3.8	97.8±2.4	96.7±3.6
22	97.1±1.9	96.9±2.0	98.1±1.3	97.1±1.9
23	98.3±0.7	98.3±0.7	99.0±0.2	98.3±0.7
24	97.5±2.2	97.4±2.3	98.2±1.7	97.5±2.2
25	98.8±1.3	98.7±1.3	99.2±0.8	98.8±1.3
26	100.0	100.0	100.0	100.0
27	99.2±0.7	99.1±0.8	99.4±0.5	99.2±0.7
28	98.3±1.4	98.2±1.5	98.9±1.0	98.3±1.4
29	99.6±0.7	99.6±0.8	99.7±0.5	99.6±0.7

30	98.3±1.9	98.2±2.0	98.9±1.3	98.3±1.9
31	98.8±1.3	98.7±1.3	99.2±0.8	98.8±1.3
32	98.8±0.0	98.7±0.0	99.2±0.0	98.8±0.0
33	97.9±1.4	97.8±1.4	98.8±0.7	97.9±1.4
34	99.2±0.7	99.1±0.8	99.4±0.5	99.2±0.7
35	98.8±0.0	98.7±0.0	99.2±0.0	98.8±0.0
36	94.6±1.9	94.3±2.0	96.3±1.5	94.6±1.9
37	99.6±0.7	99.6±0.8	99.7±0.5	99.6±0.7
38	100.0	100.0	100.0	100.0
39	98.8±1.3	98.7±1.3	99.2±0.8	98.8±1.3
40	96.3±3.8	96.1±3.9	97.8±2.3	96.3±3.8

For each subject, the mean and standard deviation of each criterion was obtained by considering 120 elements (40 movements by 3 analyzed folds).

Supplementary Table 2. The performance of the proposed ensemble classification system for each amputee subject for the detection of 40 movements; shown in mean±sd of the 40 movements by 3 analyzed folds.

**Subject number**	**(micro) F-Score**	**(macro) F-Score**	**(macro) Precision**	**(macro) Recall**
2	96.6±2.8	NaN	NaN	96.7±2.8
3	87.9±0.7	NaN	NaN	87.9±0.7
4	95.8±1.4	NaN	96.5±1.6	95.8±1.5
5	91.6±0.7	NaN	NaN	91.6±0.7

6	97.1±3.4	NaN	NaN	97.1±3.1
7	68.7±10.0	NaN	NaN	68.7±10.
8	98.3±2.6	98.2±0.7	98.8±0.5	98.3±0.7
9	97.5±1.2	97.3±1.3	98.3 ±0.8	97.5±5.0
10	74.6±5.1	NaN	NaN	74.5±1.2
11	94.2±1.9	93.8±2.1	96.7±1.5	94.1±2.0

NaN: not a number, when at least one of the movements is completely misclassified in a fold. For each subject, the mean and standard deviation of each criterion was obtained by considering 120 elements (40 movements by 3 analyzed folds).

Supplementary Table 3. The performance of the proposed features with the XGBoost and LDA classifiers (in percent) for each intact subject for the detection of 9 simultaneous movements; shown in mean±sd of the 9 movements in 4 analyzed folds.

	**XGBoost**				**LDA**			
**Subject number**	**(micro) F-Score**	**(macro) F-Score**	**(macro) Precision**	**(macro) Recall**	**(micro) F-Score**	**(macro) F-Score**	**(macro) Precision**	**(macro) Recall**
1	99.55±0.22	99.55±0.22	99.46±0.25	99.49±0.26	93.39±1.12	92.43±1.50	93.00±0.20	92.07±2.73
2	99.01±0.52	98.86±0.56	98.87±0.57	98.86±0.55	92.18±0.67	90.88±0.85	91.22±0.82	90.94±0.81
3	98.94±0.56	98.79±0.65	98.76±0.62	98.84±0.68	92.41±0.78	91.20±0.74	91.40±0.78	91.34±0.60
4	98.35±0.48	98.08±0.62	98.08±0.66	98.09±0.53	90.46±0.49	88.95±0.48	89.53±0.60	89.13±0.59
5	97.87±0.41	97.48±0.53	97.56±0.50	97.43±0.58	89.95±1.02	88.32±1.10	89.09±1.11	86.23±5.02
6	98.43±0.38	98.16±0.39	98.17±0.38	98.19±0.40	90.37±1.05	91.32±4.60	89.48±1.39	89.00±1.46
7	98.59±0.37	98.31±0.42	98.32±0.14	98.33±0.41	90.50±1.12	88.90±1.46	89.59±1.45	89.03±1.33
8	99.61±0.33	99.57±0.36	99.57±0.39	99.57±0.34	92.67±0.85	91.57±0.91	91.91±0.84	91.61±1.10
9	98.97±0.31	98.88±0.40	98.83±0.40	98.95±0.42	92.76±0.93	91.73±0.95	92.01±0.94	91.80±1.03

10	99.34±0.26	99.23±0.36	99.30±0.29	99.18±0.50	92.93±0.56	91.88±0.89	92.16±0.76	97.87±0.90
11	98.30±0.03	98.01±0.46	98.04±0.40	98.03±0.50	90.91±0.72	89.46±0.72	89.67±0.72	89.49±0.64
12	99.52±0.19	99.47±0.22	99.45±0.20	99.49±0.24	95.55±0.66	94.75±0.77	94.80±0.80	94.91±0.74
13	97.76±0.73	97.43±0.86	97.44±0.91	97.47±0.77	90.46±1.00	88.79±1.02	89.42±1.17	88.97±1.02
14	97.39±0.41	97.00±0.40	97.17±0.57	96.92±0.32	85.03±1.23	81.89±1.03	83.11±1.09	82.64±0.95
15	99.80±0.26	99.09±0.20	99.09±0.19	99.09±0.21	91.55±0.47	90.32±0.60	91.13±0.88	90.30±0.61
16	98.64±0.59	98.41±0.68	98.44±0.65	98.43±0.69	88.38±2.00	86.42±2.39	87.46±1.78	86.63±2.35
17	99.39±0.36	99.28±0.43	99.30±0.40	99.26±0.45	87.93±1.31	95.95±1.32	86.34±1.41	86.09±1.22
